# Environment width robustly influences egocentric distance judgments

**DOI:** 10.1371/journal.pone.0263497

**Published:** 2022-02-10

**Authors:** Lindsay A. Houck, Dwight J. Kravitz, John W. Philbeck

**Affiliations:** Department of Psychological and Brain Sciences, The George Washington University, Washington, DC, United States of America; Justus Liebig Universitat Giessen, GERMANY

## Abstract

Past work has suggested that perception of object distances in natural scenes depends on the environmental surroundings, even when the physical object distance remains constant. The cue bases for such effects remain unclear and are difficult to study systematically in real-world settings, given the challenges in manipulating large environmental features reliably and efficiently. Here, we used rendered scenes and crowdsourced data collection to address these challenges. In 4 experiments involving 452 participants, we investigated the effect of room width and depth on egocentric distance judgments. Targets were placed at distances of 2–37 meters in rendered rooms that varied in width (1.5–40 meters) and depth (6–40 meters). We found large and reliable effects of room width: Average judgments for the farthest targets in a 40-meter-wide room were between 16–33% larger than for the same target distances seen in a 1.5-meter-wide hallway. Egocentric distance cues and focal length were constant across room widths, highlighting the role of environmental context in judging distances in natural scenes. Obscuring the fine-grained ground texture, per se, is not primarily responsible for the width effect, nor does linear perspective play a strong role. However, distance judgments tended to decrease when doors and/or walls obscured more distant regions of the scene. We discuss how environmental features may be used to calibrate relative distance cues for egocentric distance judgments.

## 1. Introduction

The capacity of pictures to convey spatial aspects of real environments means that they can be used for planning navigation in environments before visiting them in person. Misapprehension of egocentric distances in pictured scenes (i.e., between the observer and objects pictured in the scenes) can lead to navigational errors, poor use of resources, and other negative consequences when used for this purpose. Understanding the factors that impact distance judgments in pictures thus has practical importance, in addition to the knowledge that pictures can provide about distance processing in humans more generally. Such knowledge can also improve computational vision models that aim to reconstruct 3D image properties from pictured scenes [1].

Viewers are seldom in exactly the right position relative to a photograph or picture of a scene to receive the kind of retinal stimulation that one would obtain if actually situated in the pictured environment [2–4]. Nevertheless, one can often get a compelling sense of objects in the scene appearing at different distances. The behavioral literature has often focused on issues such as the effect that viewing a picture at an angle has on shape perception [5–10]. Although relatively few studies have asked observers to judge the distance from themselves to objects in the depicted scenes, this literature has focused on issues such as the effect of camera focal length [11, 12], the effect of moving the observer closer or farther from the picture [13, 14], and the relative angular sizes of objects in the depicted scene [11, 15]. Increasing focal length is associated with not only decreased visible context but also perceptual flattening in the depth dimension [12, 16]. To some extent, changes in the size and shape of the pictured setting can mimic changes in camera focal length (e.g., smaller environments and long focal length pictures might both restrict the amount of visible context). The role of pictured environment size per se on judged distances is unclear, however, and this is the central focus of the experiments reported here.

Research on egocentric distance perception in real environments often focuses on the role of information specifying the distance to the target object itself, but there is general acknowledgement that non-target visual features can play a role in perceiving the distance of the target [17–21]. For example, for targets located beyond the effective range of primary distance cues, relative cues such as the texture gradient of the ground plane under the target can support judgments of the target distance if they are calibrated by egocentric distance information in the region close to the observer [22]. This is supported by evidence that disrupting the ground plane between the observer and the target systematically influences target distance judgments [17, 22, 23]. Beyond this, however, the role of other environmental features in egocentric distance judgments is poorly understood. Of particular relevance to the current work, there is some evidence that the size or shape of the environment can impact judgments of real-world egocentric distance [18, 20]. Quantifying this effect is both a conceptual and a methodological challenge, in that large changes in the size or shape of an environment—pictured or real—can potentially impact a host of known cues (e.g., the amount of visible ground plane texture, the availability of perspective cues, the proximity of nearby objects, the visibility of the boundaries of the environment, etc.).

In the following experiments, our approach was to study the impact of environment size and shape on distance judgments by creating computer-rendered indoor scenes of various sizes and shapes. We used crowdsourced data collection (Amazon’s Mechanical Turk, or MTurk) to collect distance judgments to traffic cone targets depicted in the scenes. Given that absolute accuracy in egocentric distance judgments within pictured scenes can vary dramatically with camera focal length [16, 24], in the experiments described below, we held focal length constant so as to focus on the role of environment size and shape. Although we will make note of the overall response accuracy when describing our results, our primary attention here is on how responses under one environmental setting compare to those in other settings, rather than how accurate they are compared to the depicted target distances. One way to characterize distance judgments is by considering both *response sensitivity*, or the degree to which responses increase as the depicted distance increases, and *bias*, or the central tendency across all responses. Response sensitivity may be thought of as the slope of the function relating the judged distance versus the depicted distance, with bias indicating the degree to which that function is shifted up or down along the vertical (response) axis. We will consider both of these aspects of performance in describing the results.

Given the known effects of image truncation and field of view in both pictured and real-world environments [12, 15, 25–28], we predicted that the size and shape manipulations would elicit systematic differences in distance judgments even when camera focal length was held constant. We found that narrow environments resulted in shorter distance judgments than wider settings. In subsequent experiments, we manipulated a variety of environmental features, including not only relatively nearby, local cues, but also more global changes in the farther reaches of the environments, in an attempt to constrain as much as possible the source of these context effects.

## 2. Experiment 1

Pictured scenes typically involve some truncation of the scene imposed by the image boundaries [28]. Increasing focal length increases this truncation, and indeed increases in both truncation and focal length are associated with decreases in distance judgments [12, 15, 28]. Similarly, limiting the field of view, even in real-world environments, can result in decreased distance judgments [25–27]. When judging distances, there may be a functional similarity between the effect of occluding parts of the environment with a visible wall (as in a small environment) and eliminating parts of the image entirely by reducing the field of view or truncating the image. If so, this predicts that smaller environments might be associated with *smaller* distance judgments, even when field of view is held constant. A central focus in Experiment 1 was to characterize the influence of the size and shape of the environment on distance judgments in pictured scenes, under conditions in which field of view and image truncation were held constant.

**Fig 1:**
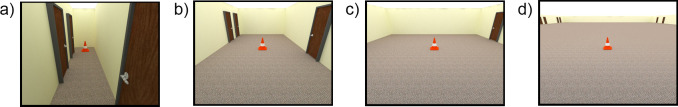
Experiment 1 stimuli examples. Depicted target distance = 4.8 m. (a) 1.5 m width, 6 m depth. (b) 5 m width, 10 m depth. (c) 10 m width, 10 m depth. (d) 40 m width, 40 m depth.

In Experiment 1, we parametrically manipulated the size and shape of environments by varying the depth (6, 10, and 40 m) and width (1.5, 5, 10, and 40 m) of simple indoor scenes. With no firm theoretical or empirical guide for choosing specific values, these values were chosen to yield a range of values on each dimension, with some oversampling of smaller values within the respective ranges. Although the specific values were chosen more by eye than by theory, we suspected that the sensitivity of distance judgments to depth and width manipulations might be greater for smaller values than for larger values, as is often the case in egocentric distance judgments when environmental context is held constant [29]. Fig 1 shows examples of four of the twelve possible combinations of these room widths and depths (Fig 1). These manipulations affect a variety of individual cues (e.g., the area of the visible ground plane, the availability of linear perspective, the visibility of nearer or farther reaches of the environment, and the proximity and visual density of environmental features to the target object). Critically, however, these manipulations did not affect the angular declination of the targets across conditions, nor any image features directly between the camera perspective and the targets themselves. This study served as a starting point for investigations targeting more specific cues in subsequent experiments.

When using crowdsourced data collection, the characteristics of the participants’ testing environment and the device used for testing are unknown. In real-world settings, even in an experiment involving rendered scenes on a computer, the characteristics of the testing computers are known and the setting is generally more well-controlled across participants. To test the generalizability of our crowdsourced data to more well-controlled settings, in Experiment 1 we compared data collected from a population of MTurk workers against a group of college students taking part in the study on a single computer in a well-controlled laboratory space. These groups differed along a number of dimensions (e.g., age, testing environment, and device used for testing), and although we had no a priori reasons to expect any of these differences to have a systematic impact on the data, this contrast allowed us to characterize any group differences that arose.

### 2.1 Method

#### 2.1.1 Participants

Participants included 108 MTurk workers and 51 undergraduate students from the Psychology participant pool at George Washington University (GW). For this and all other studies described in this paper, participants were treated in accordance with the Declaration of Helsinki. Ethics approval was given by the George Washington University Institutional Review Board. The MTurk participants were all “Master” Workers, determined by Amazon to be high performing across a wide range of tasks and who satisfy frequent statistical monitoring requirements set by Amazon. Additional requirements for participation were that workers had to be located in the United States, have a Human Intelligence Task (HIT) approval rating of 95% or above, and have completed 1000 or more HITs. The MTurk participant pool is highly diverse in terms of gender, education, age, income, and ethnicity [30]. The MTurk participants were paid $1.75 for their participation, while the GW participants were compensated with partial course credit.

#### 2.1.2 Stimuli and design

Stimuli for this and all studies described in this paper were created using SketchUp for the 3D modeling [31] and V-Ray [32] for photorealistic ray-traced rendering. Stimuli were computer-generated renderings of indoor scenes in first-person perspective (Fig 1), with the image size measuring 800 x 600 pixels. The scenes consisted of plain, beige walls and a brown carpet with a fine-grained, berber-like texture. The ceiling height was 2.7 m above the floor and was rendered as an untextured white light source across its entire surface. The height of the virtual camera was set to an average human eye height of 1.67 m, with the focal length set to 21 mm to provide visibility of the walls in the widest room condition. The camera pointed downwards 20 deg from straight ahead to provide visibility of the target’s closest distance while maintaining visibility of the ceiling and far wall in all conditions. Doors (0.91 m by 2.13 m) were placed along the left and right walls approximately every 5 m, with a pseudorandom spacing between 0.6 and 4.6 m to guard against location-specific effects in door position. The doors were intended to provide realistic elements of visual detail, while also introducing information about the scale of the environments via familiar size. Although we did not randomly place doors on every trial for each participant, we attempted to mitigate door position-specific effects by creating two possible images for each combination of condition and target distance, differing only in door locations. Each participant saw one of these two images at random when they were exposed to that combination of condition and target. A 0.69 m tall orange traffic cone with a white stripe served as the target object in each image. Data were collected via MTurk and presented with Qualtrics [33] survey software. Assuming an average viewing distance of 55 cm [34], the 800 x 600 pixel stimulus images subtended 21.8 x 16.4 degrees on average for the MTurk participants. These dimensions held true for all the GW participants. Software detected the users’ device type and did not allow participation on smartphones and tablets, thus imposing the important constraint that all participants viewed the experiment on a screen at least as large as an average laptop or desktop computer. Although screen size, display resolution and viewing distance could not be precisely controlled in the MTurk participants, dramatic departures from average viewing conditions were unlikely within the limits of usability.

Stimulus scenes had three possible room depths of 6, 10, and 40 m and four possible room widths of 1.5, 5, 10, and 40 m. While there was no theoretical basis for these particular values, they provided a range of room sizes and shapes: square or nearly square rooms of three distinct sizes, rectangular rooms varying in width and depth, and narrow hallways of various depths (Fig 1). There were 14 possible target distances (2, 2.2, 2.8, 3.2, 4.2, 4.8, 6.3, 7.3, 9.4, 10.9, 14.2, 16.3, 24.5, and 36.7 m). These distances followed two geometric progressions starting from 2.2 m, with each successive distance being 1.3 and 1.5 times the previous distance, respectively. We anticipated that in these first-person perspective images, sensitivity to changing distance might decline with increasing distance due to the spatial information being relegated to increasingly small image areas. Our method of distance selection resulted in some oversampling in the shorter distances to place more targets in the region of anticipated greater sensitivity. Our primary goal was simply to distribute targets throughout the range, however. The specific range of target distances in each image was determined by the depth of the stimulus room (6 m rooms contained distances of 2.0, 2.2, 2.8, 3.2, 4.2, 4.8 m, and 10 m rooms contained target distances of 2.0, 2.2, 2.8, 3.2, 4.2, 4.8, 6.3, 7.3, 9.4 m). Participants saw each combination of room depth, width, and target once, and all stimulus conditions and target distances were randomized within participants, for a total of 116 trials.

#### 2.1.3 Procedure

GW participants were tested individually in a quiet, empty laboratory and completed the study on a Dell Precision Tower 5810 desktop computer with an Intel i217 2.8 GHz Processor and a Dell P2213 60 Hz monitor with a resolution of 1680 x 1050, sitting approximately 55 cm from the monitor. GW participants were directed to the same online Qualtrics study as the MTurk participants through a Google Chrome web browser set to full screen. MTurk participants could access the study on any web browser of their choosing. Although the software did not control other aspects of the desktop environment (e.g., presence of other open windows), stimulus scenes had the same pixel dimensions throughout the experiment and could not be modified by the participant resizing the browser window. GW participants were monitored during the study and did not open other desktop windows while participating.

Both participant groups were given information about the purpose, methods, and potential benefits and risks of the study and gave their consent to participate before taking part in the study. All other stimuli and procedures were the same between the two groups. Consenting participants chose their preferred units of meters or feet at the beginning of the survey. Participants were instructed to view the stimuli, imagine that they were standing in the scene, and estimate the distance from their toes to the front edge of the cone. They were instructed to enter their judgment in their preferred units as a single number into a text box underneath the image. The stimuli remained on the screen until the participant responded and pressed an arrow at the bottom right of the screen to advance to the next trial. No error feedback was given. The experiment took approximately 20 minutes to complete.

### 2.2 Results

Statistics for this and all following experiments were conducted using the R statistical language with RStudio software [35]. All responses made in feet were converted to meters prior to analysis. Outlier criteria for this and all studies described in this paper were set prior to analysis to remove any trials outside 2.5 standard deviations (SD) from the mean for each combination of room condition and distance, calculated across subjects. This criterion was set based on the overall distribution of data in this first experiment. It was set to exclude obvious errors in the written judgments (e.g, “4030” feet when a participant’s responses under similar conditions were 40 or 30 feet) without trimming any real variability in the responses. Once set, the criterion was held constant throughout all the other experiment datasets and was applied prior to any subsequent analysis or evaluation. If, after this initial removal, there were any participants with less than one-third of the total trials remaining for any one room condition, that participant’s data were removed from the final dataset. For the MTurk participants, 391 trials were removed (3.12% of data) and for the GW participants, 188 trials were removed (3.2% of data). One additional participant from the MTurk group and two additional participants from the GW students were removed for having less than one-third of trials remaining in any one condition after the 2.5 SD removal, leaving 107 MTurk participants and 49 GW participants for analysis.

The data were analyzed using linear mixed models via maximum likelihood variance estimation with Satterthwaite’s method for degrees of freedom correction. The *Distance* factor was not fully crossed with the *Depth* factor, as the larger distances could not be presented in rooms with a shorter depth. We analyzed data from the three *Depth* conditions in separate analyses, in which *Width*, *Distance*, and *Group* were set as fixed factors and *Subject* as a random factor for a repeated measures design. The interactions between *Width*, *Distance*, and *Group* were also included in the model.

**Fig 2:**
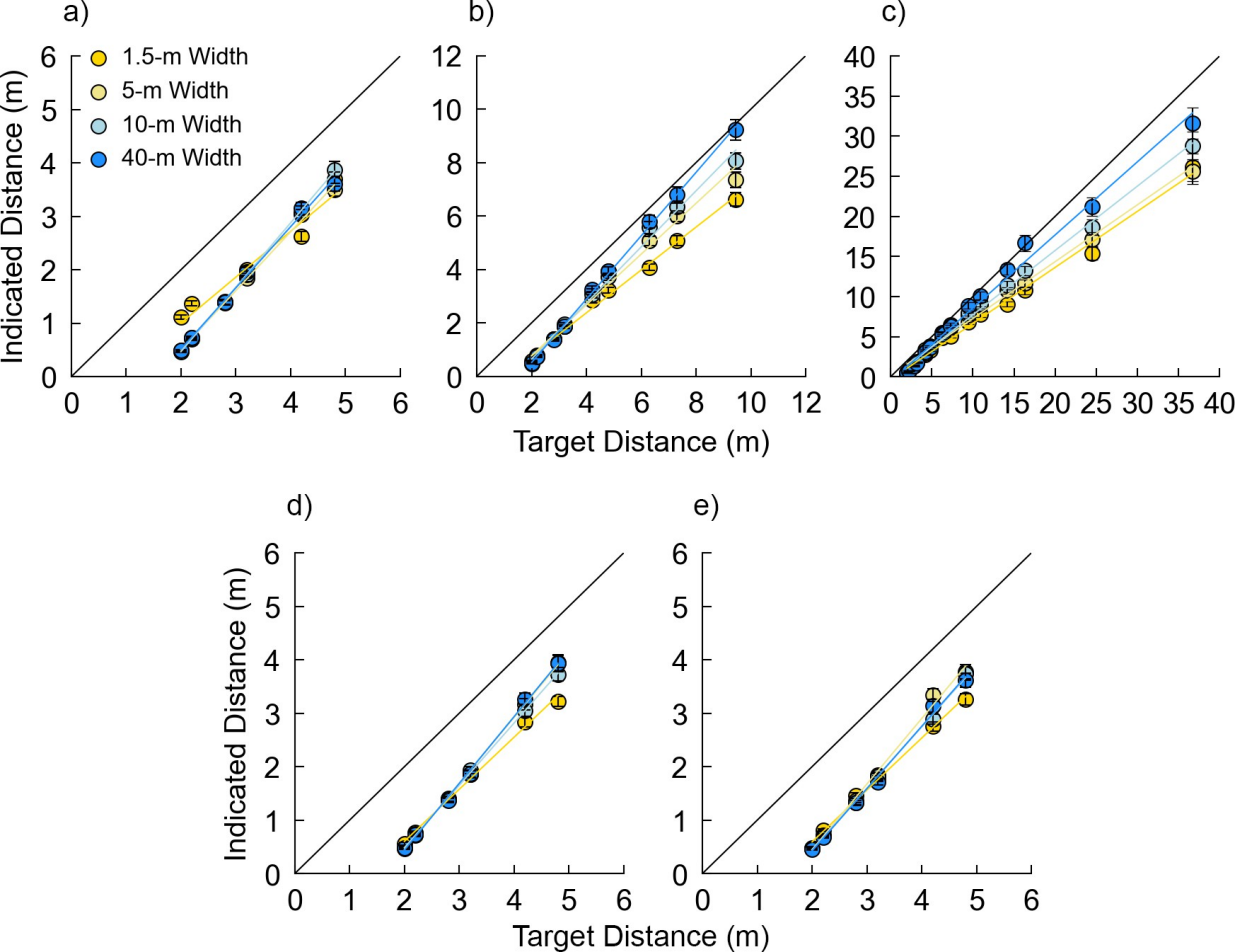
Indicated distance as a function of rendered distances in each depth condition of Experiment 1. (a) 6 m depth. (b) 10 m depth. (c) 40 m depth. (d) 10 m depth with only 2–4.8 m targets. (e) 40 m depth with only 2–4.8 m targets. Error bars denote +/- 1 standard error of the mean.

**Fig 3:**
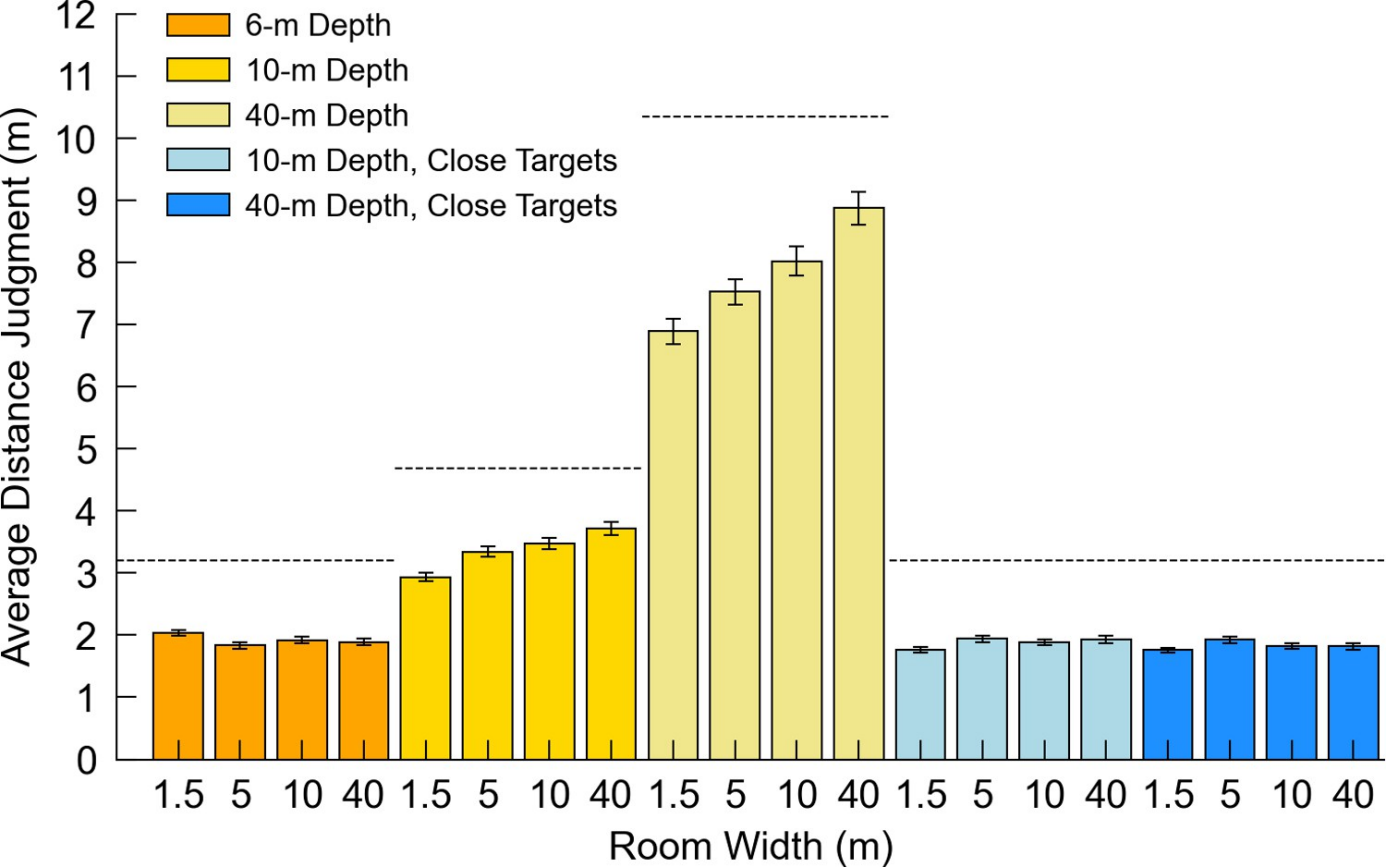
Average distance judgment for each width and depth condition in Experiment 1. The average distance judgment for each combination of *Width* and *Depth*, including the 10 and 40 m depths with only 2–4.8 m targets. The dotted lines indicate the actual average target distance for each room depth. Error bars denote +/- 1 standard error of the mean.

Fig 2 plots the indicated distance over the actual distance for all *Depth* conditions separately (Fig 2), while Tables 1 and 2 show the analysis output (Tables 1 and 2). The coefficient estimates and standard errors for all conditions are included in S5–S7 Files. Fig 3 depicts the average distance judgments across each *Depth* and *Width* condition (Fig 3). There were main effects of *Group*, *Width*, and *Distance* and significant *Width* x *Distance* interactions in all three *Depth* analyses. No other interactions were reliable in these analyses except the *Distance* x *Group* interaction. Pairwise comparisons between the *Width* conditions for each *Depth* condition are listed in Table 3. The *Width* effects indicate reliable differences in bias as a function of room width, while the *Width* x *Distance* interactions indicate that *Width*-related differences become more pronounced with increasing distance (i.e., differences in response sensitivity).

**Table 1:** Output for the depth conditions of 6 and 10 m for Experiment 1.

	6 m Depth					10 m Depth				
	*Num-DF*	*Den-DF*	*F*	*p*	*η*_*p*_^*2*^	*Num-DF*	*Den-DF*	*F*	*p*	*η*_*p*_^*2*^
**Width**	3	3502.50	9.54	<0.001	0.01	3	5329.3	42.95	<0.001	0.02
**Distance**	5	3503.0	1255.49	<0.001	0.64	8	5329.6	1276.99	<0.001	0.66
**Group**	1	152.2	6.82	0.010	0.04	1	154.0	9.58	0.002	0.06
Width*	15	3502.6	10.62	<0.001	0.04	24	5329.4	9.28	<0.001	0.04
**Distance**										
Width*	3	3502.5	0.57	0.636	<0.01	3	5329.3	2.43	0.063	<0.01
**Group**										
Distance*	5	3503.0	16.36	<0.001	0.02	8	5329.6	22.18	<0.001	0.03
**Group**										
Width*	15	3502.6	1.00	0.457	<0.01	24	5329.4	0.92	0.581	<0.01
Distance*										
**Group**										

The output and the partial eta squared values for the depth conditions of 6 and 10 m are listed here.

**Table 2:** Output for the depth condition of 40 m for Experiment 1.

	40 m Depth				
	*Num-DF*	*Den-DF*	*F*	*p*	*η*_*p*_^*2*^
**Width**	3	8374.9	28.21	<0.001	0.01
**Distance**	13	8375.3	680.95	<0.001	0.51
**Group**	1	154.6	14.39	<0.001	0.09
Width*	39	8374.9	3.50	<0.001	0.02
**Distance**					
Width*	3	8374.9	1.31	0.270	<0.01
**Group**					
Distance*	13	8375.3	34.59	<0.001	0.05
**Group**					
Width*	39	8374.9	0.24	1.0	<0.01
Distance*					
**Group**					

The output and the partial eta squared values for the depth condition of 40-m are listed here.

**Table 3:** Pairwise comparisons for *Width* for the 6, 10, and 40 m depth conditions.

	6 m Depth				10 m Depth				40 m Depth			
	1.5 m	5 m	10 m	40 m	1.5 m	5 m	10 m	40 m	1.5 m	5 m	10 m	40 m
**1.5 m**		0.20	0.12	0.14		0.41	0.54	0.78		0.64	1.13	1.99
**5 m**	<0.001		0.08	0.06	<0.001		0.13	0.37	0.011		0.50	1.35
**10 m**	0.004	0.169		0.03	<0.001	0.044		0.24	<0.001	0.011		0.85
**40 m**	0.004	0.169	0.978		<0.001	<0.001	0.004		<0.001	<0.001	0.002	

The pairwise *p*-values and their associated absolute mean differences in meters for the 6, 10, and 40 m *Depth* conditions. The *p-*values populate the lower left cells, and the absolute mean differences populate the upper right cells. Significance is determined at alpha = 0.05 with a Holm correction.

To provide a means of comparing across *Depth* conditions, we performed one additional linear mixed model analysis using only the distances that were common across all *Depth* conditions (2, 2.2, 2.8, 3.2. 4.2, and 4.8 m). We will refer to this as the *Depth Comparison* analysis. In effect, this tested the impact on distance judgments of extending the environment beyond the targets. *Width*, *Depth*, *Distance*, and *Group* were set as fixed factors and *Subject* as a random factor. The interactions between *Width*, *Depth*, *Distance*, and *Group* were also included in the model. This analysis showed main effects of *Group*, *Depth* and *Distance*, but no main effect of *Width* (Table 4). The coefficient estimates and standard errors for all conditions are included in S8 File. There were significant interactions of *Depth* x *Width*, *Depth* x *Distance*, *Width x Distance*, *Distance* x *Group*, and *Depth* x *Width* x *Distance*. We will discuss these findings in greater detail below.

**Table 4:** Output for all depth conditions for targets up to 4.8 m for Experiment 1.

	*Num-DF*	*Den-DF*	*F*	*p*	*η*_*p*_^*2*^
**Depth**	2	10,805.8	9.34	<0.001	<0.01
**Width**	3	10,805.8	1.18	0.316	<0.01
**Distance**	5	10,806.2	41656.0	<0.001	0.66
**Group**	1	154.6	6.28	0.013	0.04
Depth*Width	6	10,805.8	10.99	<0.001	<0.01
Depth*Distance	10	10,805.8	3.48	<0.001	<0.01
Width*Distance	15	10,805.8	13.48	<0.001	0.02
Depth*Group	2	10,805.8	0.66	0.514	<0.01
Width*Group	3	10,805.8	0.11	0.953	<0.01
Distance*Group	5	10,806.2	32.57	<0.001	0.01
Depth*Width*Distance	30	10,805.8	3.11	<0.001	<0.01
Depth*Width*Group	6	10,805.8	0.76	0.637	<0.01
Depth*Distance*Group	10	10,805.8	1.06	0.389	<0.01
Width*Distance*Group	15	10,805.8	0.62	0.862	<0.01
Depth*Width*Distance*Group	30	10,805.8	0.78	0.805	<0.01

The output and the partial eta squared values for all depth conditions for targets up to 4.8 m are listed here.

#### 2.2.1 Effect of group

To begin, there was a main effect of experiment group in all four analyses, with the MTurk group generally having higher distance estimates than the GW group. Across all trials, the average distance estimate for the Mturk group was 5.64 m (undershooting by 32.84% on average), while the average distance estimate of the GW group was 4.30 m (undershooting by 44.31% on average). Although additional research is required to understand the cause of this effect, the source could be due in part to systematic differences in participant characteristics [36, 37], viewing distance and/or display size [13, 15], or experimental setting. Nevertheless, no interactions involving the *Group* factor reached significance in any of the analyses except for the *Distance* x *Group* interactions. Given that there were no significant interactions between the *Group*, *Width*, and *Depth* factors in these analyses, the groups appeared to differ in terms of the overall calibration of their responses but not in terms of how they responded to variations in room depth and width.

#### 2.2.2 Effect of room depth

In the *Depth Comparison* analysis (comparing distances below 5 m across manipulations of room depth), there was a main effect of *Depth* (*p* < 0.001), with distance judgments in the 6 m depth condition generally being larger (averaging 1.89 m) than those in the 10 m and 40 m depth conditions (averaging 1.86 and 1.81 m respectively), collapsing over the GW and MTurk groups. Pairwise comparisons (two-tailed, alpha = .05 with a Holm correction) indicated that estimations in the 6 m and 40 m depths and in the 10 m and 40 m depths differed significantly (*p* < 0.001 and *p* = 0.010 respectively), though there were no differences between the 6 m and 10 m depth conditions (*p* = 0.156). Thus, the effect of extending the open space beyond the target primarily becomes apparent when there are relatively large differences in environment depth. Extending the environment depth beyond the target does not affect egocentric distance cues to the target itself, and though some work has investigated the role of relative distance cues between the observer and a target object on the perceived distance of the target itself [17, 38–40], the role of relative cues *beyond* the target is poorly understood. When binocular disparity is present, the disparity between the target and a more distant object can increase distance judgments, at least under reduced cue conditions when these are the only visible objects [41]. The effect seen here—shorter distance judgments in environments with more visible space beyond the target—is a similar pattern to what has been found before in more natural real-world environments using somewhat different methods [18, 20]. Although more work is required to explain this effect, our work here suggests that the effect itself is fairly small in pictured environments involving relatively short target distances.

#### 2.2.3 Effect of room width

For the 10 and 40 m room depth conditions, robust and unexpected effects of room *Width* were found, such that responses tended to increase with increasing room width, averaged over *Group* (Figs 2 and 3). In the 10 m room depth condition, responses averaged 2.89, 3.27, 3.40 and 3.60 m in the 1.5, 5, 10 and 40 m room width conditions, respectively; in the 40 m room depth condition, responses averaged 6.60, 7.18, 7.72 and 8.45 m in the 1.5, 5, 10 and 40 m room width conditions, respectively. The pairwise comparisons shown in Table 3 indicate that these increases in distance judgments as a function of room width were generally significant between the different levels of *Width*. In the 6 m room depth condition, the pattern differs somewhat. Although there was again a main effect of *Width*, here the narrowest room was associated with larger judgments: responses averaged 2.00, 1.80, 1.87 and 1.88 m in the 1.5, 5, 10 and 40 m room width conditions, respectively. Importantly, however, for the 6 m depth condition, the images of the 10 and 40 m wide rooms were indistinguishable for each target distance, as the walls and doors to the left and right were out of the frame and not visible. One would thus not expect width-related response differences in these conditions. In the *Depth Comparison* analysis, there was no main effect of *Width*, with responses averaging 1.83, 1.87, 1.85 and 1.85 m in the 1.5, 5, 10 and 40 m width conditions, respectively. Note that these means are somewhat different than in the 6 m depth condition analysis, because they incorporate judgments of the shorter distances from the 10 and 40 m room depth conditions. Although there was a significant *Depth* x *Width* interaction, this is difficult to interpret due to the indistinguishable images in the 10 and 40 m width rooms for the 6 m depth condition.

### 2.3. Discussion

The room width effects in the 10 and 40 m room depth conditions are surprising for two reasons. One is that the width dimension is orthogonal to the depth dimension, and one might initially assume that manipulating width would do nothing to alter the cues relevant for perception in the orthogonal depth dimension. It is certainly true that altering room width does not impact the primary egocentric cues specifying the distance of the target object itself (e. g., angular declination). Altering room width does, however, impact a variety of features surrounding the target object, which might play a role in influencing the perceived target distance. For example, as the room width becomes narrower, surrounding objects are brought in closer proximity to the target. This provides denser gradients of both linear perspective and texture (via texture elements along the walls) [19, 42–45]. Importantly, this highlights a second reason why the width effect is surprising: denser cue gradients might be expected to *increase* distance judgments in the narrow room conditions, but in fact the opposite was true in Experiment 1 –these conditions were reliably associated with *smaller* distance judgments, with the judgments increasing systematically as the walls to the left and right moved farther away.

The visual features of the environments that underlie this pattern are not clear. Manipulating room width changes a variety of features. For example, narrow rooms (or hallways) typically have walls that occlude much of the ground plane. Wu and colleagues [46] have suggested that the nearby ground plane provides a reliable source of egocentric distance information in real-world environments that is used to calibrate relative distance cues (given by texture gradients, linear perspective, and so forth) in successively farther reaches of the environment. In support of this view, they have shown that interrupting the nearby ground plane in front of a target can lead to shorter distance judgments than when the ground plane is uninterrupted [17]. Occluding the ground plane with walls, then, might play a role in the smaller distance judgments in our narrow room (hallway) conditions. The walls in narrow hallways also occlude much of the farther reaches of the environment. This could limit the availability or reliability of global image features (e.g., the far wall or farther reaches of the ground plane) that could be used in estimating the overall scale of target distances throughout the observable range [47]. Much of the past work studying the impact of ground plane texture discontinuities on egocentric distance judgments have involved discontinuities lying directly between the observer and the target [17, 38, 40]. As discussed above, this is thought to impact distance judgments by interrupting integration of relative distance cues across the ground surface [46]. In our case, the ground texture discontinuities imposed by walls are displaced laterally from this axis, possibly leaving such integration processes intact. This is a potentially meaningful distinction. Even so, walls introduce large discontinuities in the overall ground plane texture and this could influence distance judgments in some way. We will continue to explore this issue in the other experiments described below.

In our images, the walls were lined with familiar-sized objects—doors. The narrow room (hallway) images generally contained more visible doors than the wider room images. The doors were also generally larger in terms of visual angle when they were in the foreground and were concentrated more densely around the center of the image. It is not yet clear whether these image features play a role in the room width effect. One other image feature of narrow rooms is that the horizontal intersection line between the far wall and the ground plane is shorter, owing to the narrow width of the room—this could impair estimation of the horizon and perhaps also of the viewer’s eye height. The patterns of linear perspective in the images also differ substantially between narrow and wide rooms, with both the angles and proximity of linear segments varying across room widths. This discussion does not exhaust the list of features that change systematically with room width, and many of these features are not cleanly separable. Nevertheless, in the remaining experiments in this article we will test various combinations of these features in an attempt to shed light on the visual information underlying the large changes in egocentric distance judgments across environment widths.

## 3. Experiment 2

**Fig 4:**
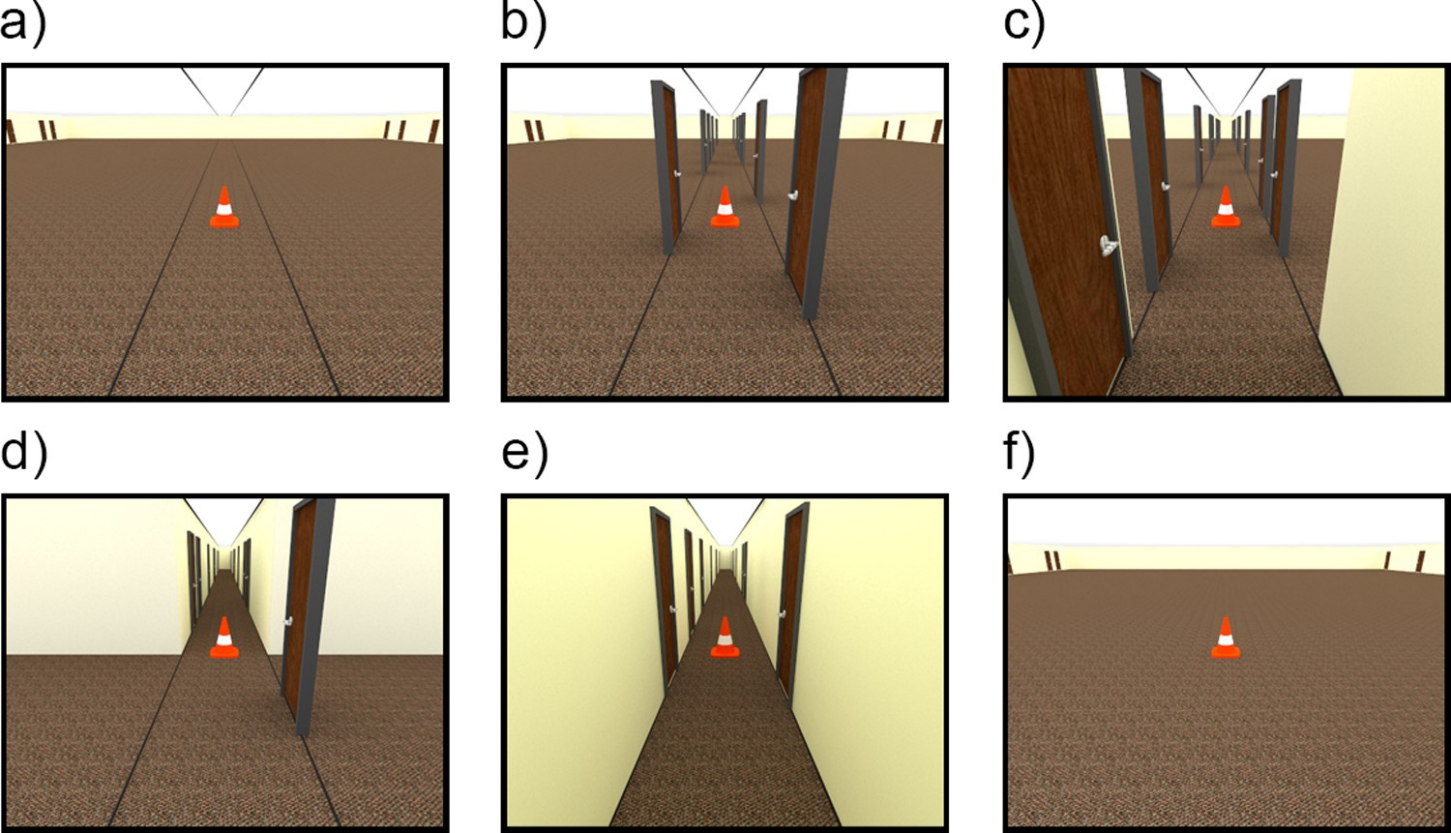
Example stimuli from Experiment 2. Depicted target distance = 4.8 m. (a) Linear Perspective Lines. (b) Freestanding Doors. (c) Near Ground Occluded. (d) Far Ground Occluded. (e) Narrow (1.5 m width). (f) Wide (40 m width).

Experiment 1 showed that wider environments resulted in larger distance judgments, but the cue bases for the effect remain unknown. Of the many features that differ between wide and narrow pictured environments, one goal of Experiment 2 was to test the role of linear perspective in producing the width effect. The linear perspective information was less strong in the wide environment in Experiment 1 than in the narrow environment, as both the walls and doors in the wide environment were displaced to the far periphery. Experiment 2 included two conditions to test the effect of linear perspective: 1) In the *Linear Perspective Lines* condition (Fig 4A), dark lines were added on the floor and ceiling of the *Wide* room to mark the location where the walls would appear in the *Narrow* room. Like the *Wide* rooms, this condition provided full view of the ground plane, the far environment boundaries, and the visible horizon, but with enhanced linear perspective. 2) In the *Freestanding Doors* condition (Fig 4B), the walls in the *Narrow* condition were removed, but the doors and linear perspective lines were retained. The doors enhanced the linear perspective information still further and yielded a condition that was well-matched to the *Narrow* condition in Experiment 1 in terms of the overall availability of linear perspective. The primary difference was that the *Freestanding Doors* condition provided more visibility of the ground plane, far environment boundaries and visible horizon than Experiment 1’s *Narrow* condition. If linear perspective plays a substantial role in producing the room width effect, we predicted that distance judgments in these conditions would be smaller and more similar to those obtained in the *Narrow* room than the *Wide* room. By contrast, if general visibility of the ground plane, environment boundaries and visible horizon play a role, both the *Linear Perspective Lines* and *Freestanding Doors* conditions should yield the larger distance judgments associated with the *Wide* room condition, because all these conditions provide visibility of those features (albeit with somewhat less visibility in the *Freestanding Doors* condition due to occlusion by the doors).

A second goal of Experiment 2 was to contrast the visibility of the relatively nearby ground plane versus visibility of the farther reaches of the environment. There is evidence that in both real-world and pictured environments, disruption or occlusion of the nearby ground plane can reduce distance judgments [17, 38, 40]. These studies have generally disrupted the ground plane directly between the observation point and the target; narrow hallways leave this space unobstructed, although the walls do occlude vision of the more lateral regions of the nearby ground plane and this occlusion could play a role in producing the width effect. Another view is that visibility of more global features in the farther regions of the scene could play a role in producing the width effect. Image features associated with the overall scene context have been shown to support judgments that group scenes into broad “mean depth” categories—e.g., whether the scene takes up kilometers of space or only a few meters of space [47, 48]. Global image features, for example, those associated with the farthest spatial boundaries or the openness of the space, could influence egocentric distance judgments by calibrating the available relative distance cues. To contrast the role of near versus far image features, Experiment 2 included two conditions that selectively occluded portions of the pictured scenes. In the *Near Ground Occluded* condition (Fig 4C), the nearby portion of the room resembled the *Narrow* condition from Experiment 1, occluding the lateral portions of the nearby ground plane, while the farther reaches of the space were left open except for a narrow corridor of freestanding doors. In the *Far Ground Occluded* condition (Fig 4D), there was a narrow hallway in the farther reaches of the environment, thus occluding the farther ground plane and leaving the nearby ground plane open except for several freestanding doors. If visibility of near versus far regions of the space plays a role in producing the room width effect, we predicted that this would be manifested in the contrast between these two conditions, such that larger distance judgments would indicate the condition that provides visibility of the crucial part of the scene, with smaller distance judgments indicating the condition that occludes the crucial part of the scene.

### 3.1 Method

#### 3.1.1 Participants

Participants consisted of 98 “Master” MTurk Workers. Ethics approval and ethical treatment of participants were as described in Experiment 1.

#### 3.1.2 Stimuli and design

All stimuli were rendered with a room depth of 40 m and 12 target distances (4.2, 4.8, 6.3, 7.3, 9.5, 10.9, 14.2, 16.3, 20, 24.5, 30, and 36.7 m) from the observer’s viewpoint. Given room width-related response differences in Experiment 1 were more apparent in the farther distances, here we eliminated some of the nearest distances (3.2 m and below) and sampled more densely in the farther region by adding targets at 20 and 30 m. A traffic cone was the target. For this and all subsequent experiments, the image size measured 500 x 375 pixels. The decrease in size from Experiment 1 was motivated by feedback from some participants that the stimuli took too long to load. Although we doubted that the larger image size was critical for producing the width-related effects in Experiment 1, Experiment 2 provided a means of testing whether the effect would replicate with smaller images. Six room conditions were included in the design: 1) The *Linear Perspective Lines* condition was identical to the *Wide* room condition in Experiment 1, but with dark lines added to the floor and ceiling marking the boundaries of the 1.5 m narrow hallway. 2) The *Freestanding Doors* condition also included these linear perspective lines but added a narrow corridor of freestanding doors marking the boundaries of a 1.5 m narrow hallway. 3) The *Near Ground Occluded* condition included narrow walls separated laterally by 1.5 m and extending 2.0 m into the room from the camera position, occluding the near ground and leaving the far ground visible. 4) The *Far Ground Occluded* condition included narrow walls, separated by 1.5 m, beginning 5 m into the room from the camera position and extending laterally off-screen in both directions, occluding the far ground and leaving the near ground visible. Both occlusion conditions included freestanding doors marking the boundaries of a 1.5 m narrow hallway outside of the occluded portion of the room. Lastly, 5) the *Wide* (40 m room width) and 6) *Narrow* (1.5 m room width) conditions from Experiment 1 were also included. All stimuli conditions and target distances were randomized within participants for a total of 72 trials.

#### 3.1.3 Procedure

The study was executed as described in Experiment 1.

### 3.2 Results

**Fig 5:**
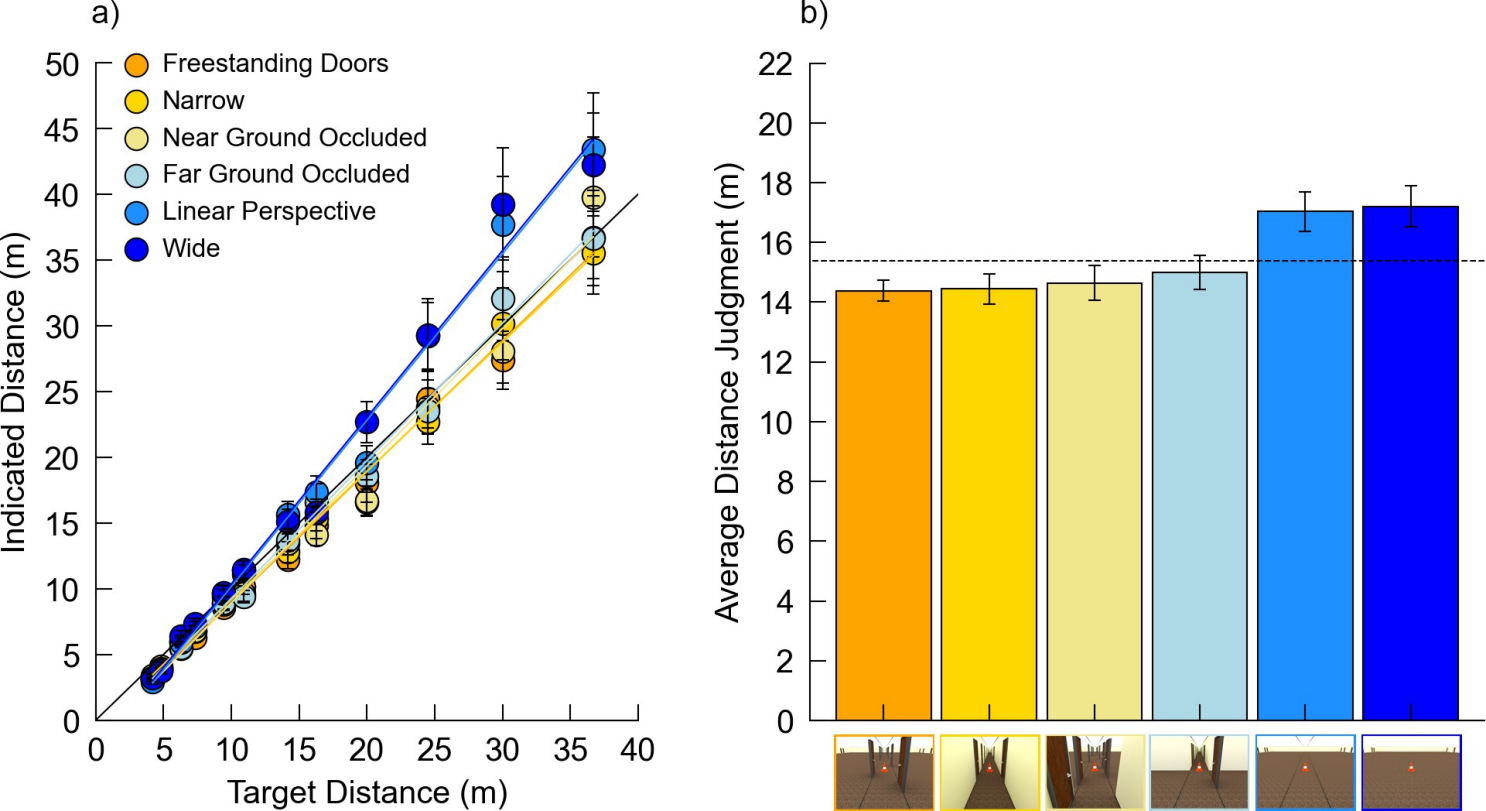
Data from Experiment 2. Error bars denote +/- 1 standard error of the mean. (a) Plot of the indicated distance over the actual distance for all conditions. (b) Average distance judgment for each room condition. The dotted line indicates the actual average target distance. Images on the x-axis depict the room width conditions from Fig 4.

218 trials fell outside 2.5 SD from the mean for each combination of room condition and target and were removed (3% of data). One participant was removed because fewer than one-third of the trials remained in one or more conditions after 2.5 SD outlier removal, leaving 97 participants in the final analysis. Fig 5A plots the indicated distance over the actual distance for each combination of room condition and target distance and Fig 5B shows the average distance judgment for each condition (Fig 5). The judgments undershot the physical (rendered) distances by only 4.79% on average, which was less than the 32.84% undershooting for the MTurk participants in Experiment 1. Experiment 1 utilized somewhat larger images–a feature known to reduce distance judgments in pictures [14], so this may play a role in explaining the calibration differences between experiments. Although we will comment on calibration differences across studies when appropriate, a full exploration of these differences is beyond the scope of the current paper.

The data were analyzed using linear mixed models via maximum likelihood variance estimation with Satterthwaite’s method for degrees of freedom correction. *Room* and *Distance* were set as fixed factors with *Subject* as a random factor for a repeated measures design. The interactions between *Room* and *Distance* were also included in the model. The results indicated a main effect of *Room* (*F*[5,6705.0] = 9.15, *p <* 0.001, *η*_*p*_^*2*^ < 0.01), a main effect of *Distance* (*F*[11,6705.3] = 425.55, *p <* 0.001, *η*_*p*_^*2*^ = 0.41), and a significant *Room* x *Distance* interaction (*F*[55,6705.0] = 1.72, *p <* 0.001, *η*_*p*_^*2*^ = 0.01). Here, the main effect of *Room* indicates significant differences in the response bias for each room condition, and the *Room* x *Distance* interaction represents significant differences in response sensitivity, or the slopes of the functions relating estimated to actual target distance. Pairwise comparisons (at alpha = 0.05 with a Holm correction) showed significant differences in bias between the *Wide* room and all other rooms except *Linear Perspective*, which was also significantly different from all other rooms (Table 5). The coefficient estimates and standard errors for all conditions are included in S9 File.

**Table 5:** Pairwise comparisons and absolute mean differences for Experiment 2.

	Freestanding Doors	Narrow	Near Ground Occluded	Far Ground Occluded	Linear Perspective	Wide
**Freestanding Doors**		0.06	0.22	0.61	2.64	2.82
**Narrow**	1.0		0.20	0.55	2.59	2.76
**Near Ground Occluded**	1.0	1.0		0.35	2.38	2.56
**Far Ground Occluded**	1.0	1.0	1.0		2.04	2.21
**Linear Perspective**	*<*0.001	*<*0.001	<0.001	0.005		0.18
**Wide**	*<*0.001	<0.001	*<*0.001	0.004	1.0	

The pairwise *p*-values and their associated absolute mean differences in meters for Experiment 2. The *p-*values populate the lower left cells, and the absolute mean differences populate the upper right cells. Significance is determined at alpha = 0.05 with a Holm correction.

### 3.3 Discussion

The main effect of *Room* indicates that average bias in responses differed between room conditions, while the significant *Room* x *Distance* interaction confirms suspicions from Experiment 1 –that the *Room* conditions are further distinguished by differences in response sensitivity, such that judgments in wider rooms become increasingly larger as target distances increase. The main effect of *Distance* is unsurprising, as it indicates a difference in estimated distances based on the actual distance of the target. The width effect found in Experiment 1 was replicated here, with distance judgments being significantly larger in the *Wide* room compared to the *Narrow* room. This held true despite the slightly smaller image size compared to Experiment 1. The *Linear Perspective Lines* condition added lines on the floor and ceiling to the *Wide* condition images, and this manipulation did not result in significant differences in response bias compared with the *Wide* condition. This suggests that the linear perspective provided by the walls in the *Narrow* condition is not by itself a major factor in eliciting the effect of room width in distance judgments. Interestingly, adding freestanding doors to these linear perspective lines had a dramatic effect, with responses now displaying the shorter distance judgments associated with *Narrow* hallways rather than the longer judgments associated with *Wide* rooms. This occurred despite the fact that removing the walls exposed more of the ground plane in both nearby and farther regions, as well as more of the far boundaries of the environment. This indicates that the visibility of these features does not play a strong role in producing the *Wide* pattern—at least when the doors are present. Given this result, it is perhaps not surprising that the *Near-* and *Far Ground Occluded* conditions also did not differ significantly from the *Narrow* room pattern, because both of those conditions included doors extending the length of the hallway. A more targeted test of the role of occluding near or far parts of the environment would be to remove the doors in the open (unoccluded) parts of the image. We will test this in Experiment 3.

In sum, the freestanding doors apparently play a role in eliciting the smaller distance judgments associated with *Narrow* environments. Adding doors has several effects, including adding to the available linear perspective, adding two planes of texture gradients as the lines of doors extend into depth, and adding objects near the possible target locations. It is not clear what aspect of the doors contributes to the width effect. We will return to this issue in Experiments 3 and 4.

## 4. Experiment 3

In Experiment 2, the *Near-* and *Far Ground Occluded* conditions, as well as the *Freestanding Door* condition, yielded a pattern of smaller distance judgments that is characteristic of the *Narrow* environments. The relative role of occluding near versus far regions of the environment, however, is difficult to determine from the results of Experiment 2, because both of these conditions contained doors, either freestanding in regions where the ground was otherwise visible, or embedded in walls. The freestanding doors by themselves were sufficient to produce the *Narrow* pattern in Experiment 2, so that study leaves unsolved the question of whether the width effect comes more from features specific to the doors, or whether occlusion of the environment (possibly restricted to near or far regions) drives the effect. We have already discussed reasons why occlusion could be a contributing factor. Doors, however, could play a distinct role by way of providing a richer cue environment: they enhance linear perspective and texture gradients, they provide reference objects in the vicinity of the target, and they are familiar-sized objects that could be used to provide a sense of scale. If the width effect disappears when the doors are removed, this would help constrain the search for cue bases of the width effect. Experiment 3 addressed this issue by removing the doors in all conditions. It included trials based on the *Wide* and *Narrow* conditions from Experiments 1 and 2, but without doors. Given that these experiments showed robust differences between the *Wide* and *Narrow* conditions, there is good reason to expect a similar effect here.

To further assess the role of occluding parts of the environment on the room width effect, Experiment 3 manipulated occlusion in four additional conditions: 1) a *Near Ground Occluded* condition that matched the condition from Experiment 2; 2) a close-range *Far Ground Occluded* condition that matched the condition from Experiment 2; 3) an intermediate *Far Ground Occluded* condition that occluded the mid-range of the environment and beyond; and 4) a farther *Far Ground Occluded* condition that occluded only the farthest range of the environment. One possible outcome is that when the doors are removed, responses are similar across all tested conditions. This would clearly implicate features associated with the doors in producing the width effect and would rule out explanations associated with occluding different parts of the environment. Another possibility is that, in the absence of doors, the *Narrow* pattern of responses is elicited when only the nearby environment or only the far environment is occluded. This kind of outcome would be informative about which region plays a role in the width effect.

Regarding the 3 levels of occlusion of the farther portions of the environment, one might predict a graded response, such that as progressively more of the far environment is occluded, responses increasingly resemble the *Narrow* pattern. Although perhaps less diagnostic from the perspective of specifying what visual features are responsible for the width effect, such a result would confirm that the presence of doors is not required to elicit the *Narrow* pattern and that the effect is driven by some feature that varies in conjunction with occlusion of the farther environment. Another possible outcome is that the *Narrow* pattern is elicited in all three *Far Occlusion* conditions. This would be striking, given the very salient differences in the appearance of these settings and the visual similarity between the farthest *Far Occlusion* condition and the *Wide* condition images. Such a result would focus attention on aspects that these three conditions hold in common with the *Narrow* room images. We will return to this question in the Experiment 3 discussion.

### 4.1. Method

#### 4.1.1 Participants

Participants consisted of 101 Master MTurk Workers. Participants here were paid $2.50 in exchange for their participation. This experiment was conducted at a time when other studies were in progress in the laboratory, and the current study was posted at the compensation rate for those slightly longer experiments. The difference in compensation compared to the other studies in this article is likely to be of little consequence, however.

#### 4.1.2 Stimuli and design

**Fig 6:**
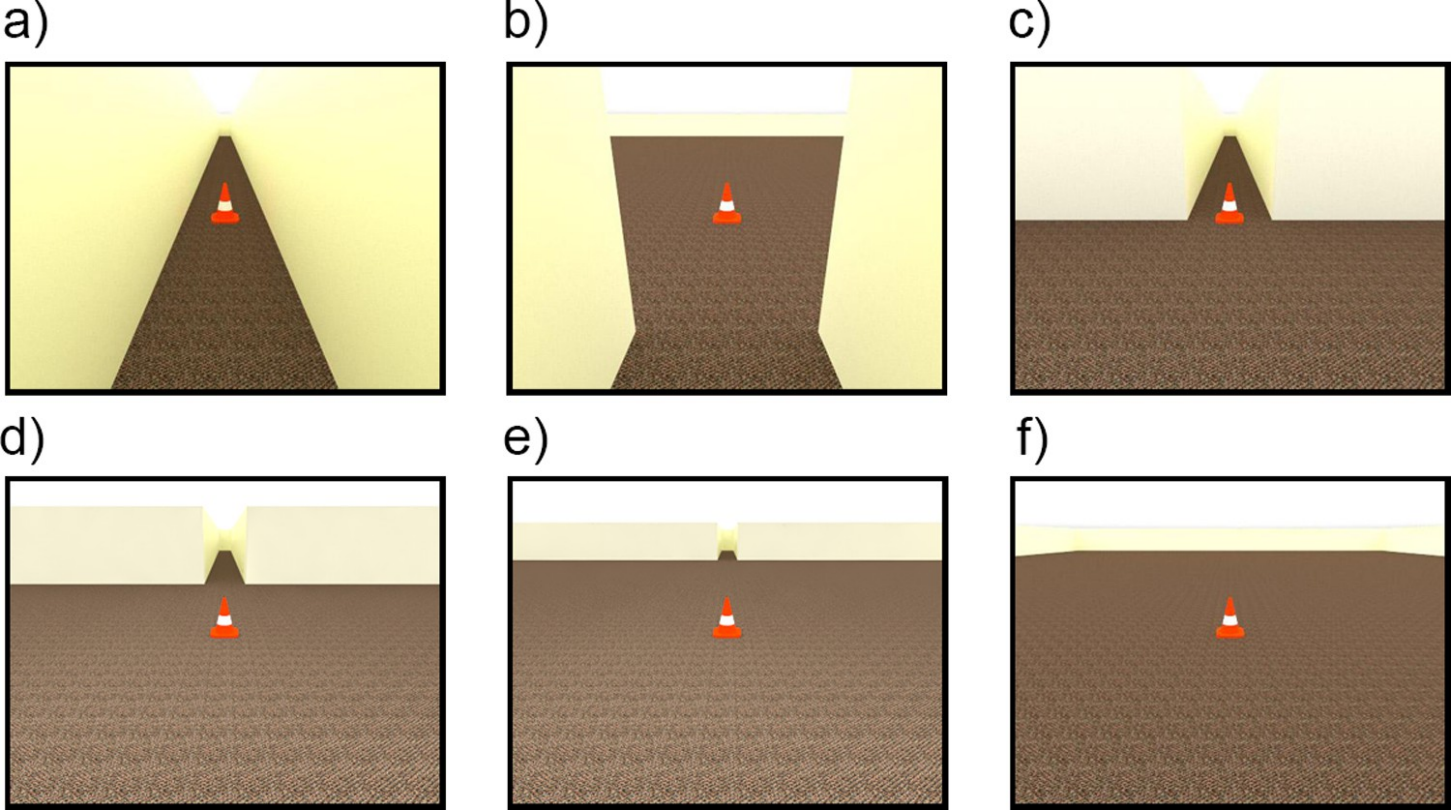
Example stimuli from Experiment 3. Depicted target distance = 4.8 m. (a) Narrow. (b) Near Ground Occluded. (c) Far Ground Occluded–Close. (d) Far Ground Occluded–Mid. (e) Far Ground Occluded–Far. (f) Wide.

All stimuli were rendered with a room depth of 40 m and the same 12 target distances as Experiment 2. Images were 500 x 375 pixels. In contrast to the previous two experiments, no doors were included in any of the stimulus scenes. Six conditions were used in Experiment 3, as depicted in Fig 6: 1) *Narrow* (1.5 m width) (Fig 6A); 2) *Near Ground Occluded*, with narrow walls separated laterally by 1.5 m and extending 2.0 m into the room from the camera position (Fig 6B); 3) *Far Ground Occluded-Close*, with walls beginning 5 meters into the room and extending laterally, leaving a 1.5 m hallway beyond (Fig 6C); 4) *Far Ground Occluded-Mid*, with walls beginning 11 meters into the room and extending laterally, leaving a 1.5 m hallway beyond (Fig 6D); 5) *Far Ground Occluded-Far*, with walls beginning 23 meters into the room and extending laterally, leaving a 1.5 m hallway beyond (Fig 6E); and 6) *Wide* (40 m width) (Fig 6F). The *Narrow*, *Near Ground Occluded*, *Far Ground Occluded-Close*, and *Wide* conditions were the same as in Experiment 2, except with no doors. All stimulus conditions and target distances were randomized within participants for a total of 72 trials.

#### 4.1.3 Procedure

The study was executed as described in Experiment 1.

### 4.2 Results

Prior to outlier removal, two participants were removed from the dataset because they gave the same number for every trial. Of the remaining 99 participants, 209 trials fell outside 2.5 SD from the mean for each combination of room condition and target and were removed (2.93% of data). One additional participant was removed for having less than one-third of the trials remaining in one or more condition after the 2.5 SD removal, leaving 98 participants in the final analysis.

**Fig 7:**
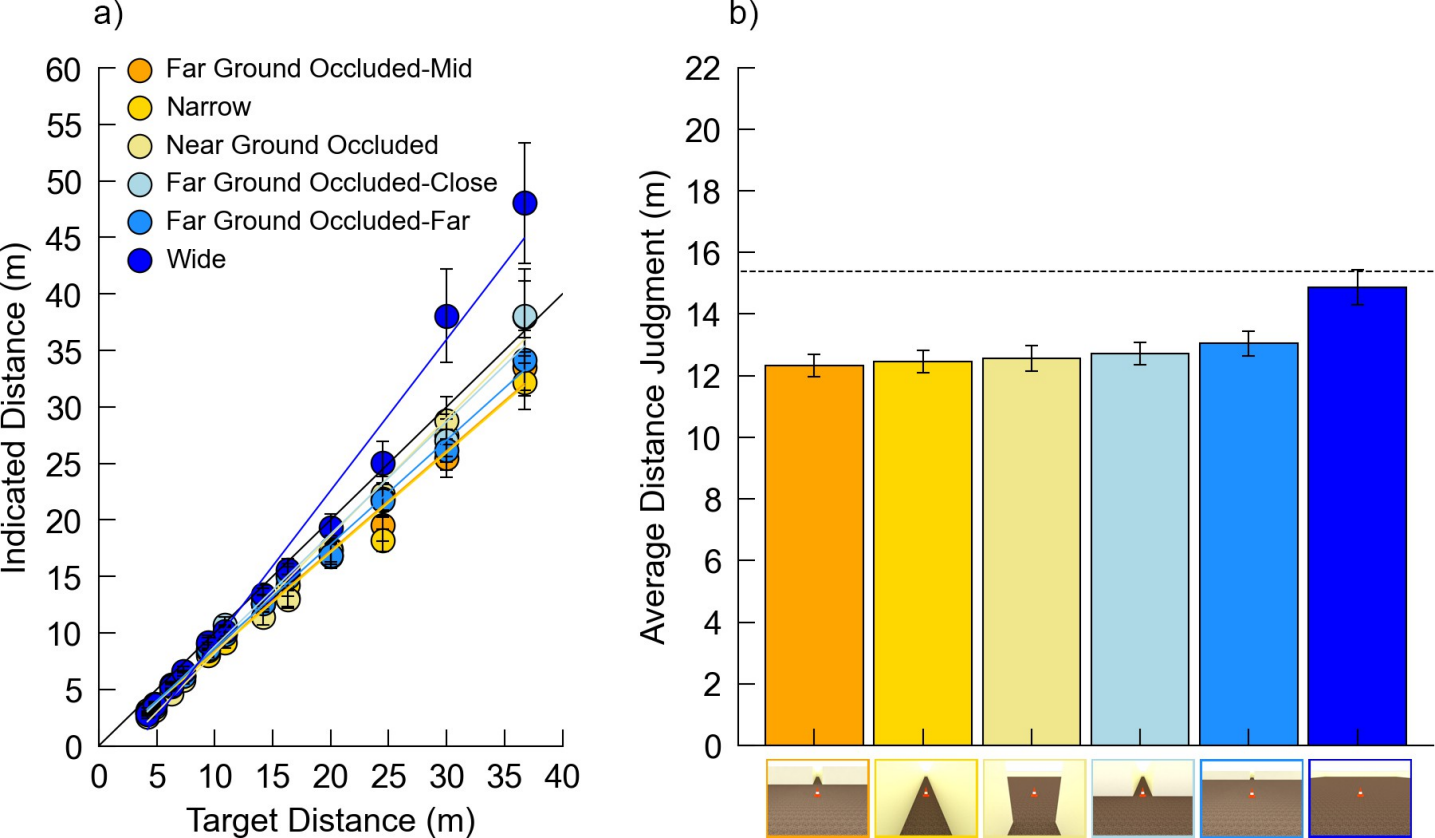
Data from Experiment 3. Error bars denote +/- 1 standard error of the mean. (a) Plot of the indicated distance over the actual distance for all conditions. (b) Average distance judgment for each room condition. The dotted line indicates the actual average target distance. Images on the x-axis depict the room width conditions from Fig 6.

Fig 7A plots the indicated distance over the actual distance for each combination of room condition and distance and Fig 7B shows the average distance judgment for each condition (Fig 7). In this study, the judgments undershot the physical (rendered) distances by 13.28% on average. The data were analyzed using linear mixed models via maximum likelihood variance estimation, with Satterthwaite’s method for degrees of freedom correction. *Room* and *Distance* were set as fixed factors and *Subject* as a random factor for a repeated measures design. The interactions between *Room* and *Distance* were also included in the model. The results indicated a significant main effect of *Room* (*F*[5,6796.6] = 13.28, *p* < 0.001, *η*_*p*_^*2*^ = 0.01), a significant main effect of *Distance* (*F*[11,6796.7] = 499.33, *p* < 0.001, *η*_*p*_^*2*^ = 0.45), and a significant *Room* x *Distance* interaction (*F*[55,6796.5] = 2.94, *p* < 0.001, *η*_*p*_^*2*^ = 0.02), mirroring the results from Experiment 2. Pairwise comparisons (at alpha = 0.05 with a Holm correction) showed significant differences in response bias between *Wide* and all other room conditions. No other comparisons were significantly different (Table 6). The coefficient estimates and standard errors for all conditions are included in S10 File.

**Table 6:** Pairwise comparisons and absolute mean differences for Experiment 3.

	Far Ground Occluded-Mid	Narrow	Near Ground Occluded	Far Ground Occluded-Close	Far Ground Occluded- Far	Wide
**Far Ground Occluded-Mid**		0.14	0.25	0.40	0.73	2.55
**Narrow**	1.0		0.11	0.27	0.59	2.41
**Near Ground Occluded**	0.906	0.906		0.16	0.48	2.30
**Far Ground Occluded- Close**	0.522	0.522	1.0		0.32	2.15
**Far Ground Occluded- Far**	1.0	1.0	1.0	1.0		1.82
**Wide**	<0.001	<0.001	<0.001	<0.001	<0.001	

The pairwise *p*-values and their associated absolute mean differences in meters for Experiment 3. The *p-*values populate the lower left cells, and the absolute mean differences populate the upper right cells. Significance is determined at alpha = 0.05 with a Holm correction.

### 4.3 Discussion

As seen in Experiment 2, here, the main effect of *Room* indicates a difference in response bias across the various room conditions, and the *Room* x *Distance* interaction indicates that the *Room* effect is associated with differences in response sensitivity, with judgments in wider rooms becoming increasingly larger as target distances increase. The *Narrow* condition again elicited systematically shorter distance judgments than the *Wide* condition. This is notable in that these images did not contain any of the features associated with doors that might have contributed to the room width effect in Experiments 1 and 2. Interestingly, responses in all other conditions were statistically similar to those in the *Narrow* condition and to each other. This suggests two things: 1) the presence of doors in Experiment 2 is not necessary to elicit the width effect, and 2) occluding the environment, in either nearby or farther regions, decreases distance judgments similarly to the pattern in *Narrow* rooms.

Many of our manipulations so far have had the effect of making more distant parts of the scene less visible. Certainly, the presence of walls or doors in the scene occludes regions behind those features. Another feature of these manipulations, however, is that they put objects (doors and/or walls) in the vicinity of the targets. These objects might play a role by engaging attention in a different way or by enhancing the constellation of relative cues in the vicinity of the target. Thus, in principle, the presence of objects could be the primary factor underlying the width effect, rather than occlusion of more distant regions of the environment. Testing this would entail including objects that do not occlude much of the environment. The *Freestanding Doors* condition of Experiment 2 partially achieved this, in that the absence of doors left much of the environment visible, particularly in the farther reaches of the scene. In keeping with a possible role of objects per se, this condition yielded the *Narrow* response pattern. Doors in the more nearby regions in this condition did still occlude substantial portions of the more distant environment, however. Experiment 4 provides a stronger test of the idea that objects near the targets, rather than occlusion of the farther environment, are primarily responsible for the width effect.

## 5. Experiment 4

Experiment 3 suggested that the presence of objects (walls) in the vicinity of the target locations plays a strong role in eliciting the shorter distance judgments characteristic of *Narrow* settings. That study manipulated the location of walls near the targets in the three *Far Occlusion* conditions and found that all three conditions yielded judgments that resembled the *Narrow* response pattern. The *Far Occlusion* conditions, however, not only placed objects (walls) in the vicinity of the target locations, but also occluded parts of the farther environment, including the far ground plane and large portions of the farthest wall of the rooms. One aim of Experiment 4 was to test the unique role of objects near the farther target locations under conditions of minimal ground plane occlusion.

In Experiment 4, we included doors in all conditions, as these are objects that do not occlude large portions of the environment, particularly when they appear in the farther regions. We contrasted a condition in which the doors appeared only in the foreground (*Near Doors*) against a condition in which there were freestanding doors only in the farther regions of the space (*Far Doors*). For comparison, a full *Freestanding Doors* condition included freestanding doors along the full length of the space.

A fourth condition in Experiment 4 used the wide room, but with all ground plane texture removed (*No Ground Texture)*. If various degrees of occlusion of the ground plane texture was responsible for the decreased distance judgments (whether due to the presence of doors or walls), removing the ground texture entirely should yield decreased judgments even in the wide-room environment. Two other conditions included the standard *Narrow* and *Wide* rooms with doors, yielding 6 total conditions.

### 5.1. Method

#### 5.1.1 Participants

Participants consisted of 101 Master MTurk Workers. Participants here were paid $1.75.

#### 5.1.2 Stimuli and design

**Fig 8:**
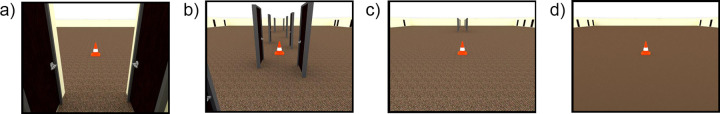
Example stimuli from Experiment 4. Depicted target distance = 4.8 m. (a) Near Doors. (b) Freestanding Doors. (c) Far Doors. (d) No Ground Texture. Not pictured: Narrow and Wide conditions, which were the same stimuli as in Experiment 2.

All stimuli were rendered with a room depth of 40 m and the same 12 target distances from Experiments 2 and 3. Images were 500 x 375 pixels. Three conditions manipulated the location of the doors and consequently tested the visible objects and boundaries in proximity to the target: 1) *Near Doors* (Fig 8A); this condition included two doors in the immediate foreground separated laterally by 1.5 m. These doors were embedded in walls in the same locations as the walls in the *Near Ground Occluded* conditions of Experiments 2 and 3, although here, only a thin sliver of the walls themselves was visible outside of the doors. In these locations, the doors did occlude some of the nearby ground plane but provided a salient connection to the doors in the *Freestanding Door* and *Far Door* conditions. 2) *Freestanding Doors* along the boundary of a narrow hallway extending the full depth of the room (Fig 8B); and 3) *Far Doors*, which included freestanding doors along the boundary of a narrow hallway just near the far targets (Fig 8C). A *No Ground Texture* (Fig 8D) condition was also included, which removed visible ground texture from the *Wide* room condition. The *Wide* (40 m wide room) and *Narrow* (1.5 m wide) rooms from the previous experiments were also included. All stimuli conditions and target distances were randomized within participants for a total of 72 trials.

#### 5.1.3 Procedure

This study was conducted as described in the previous experiments.

### 5.2 Results

**Fig 9:**
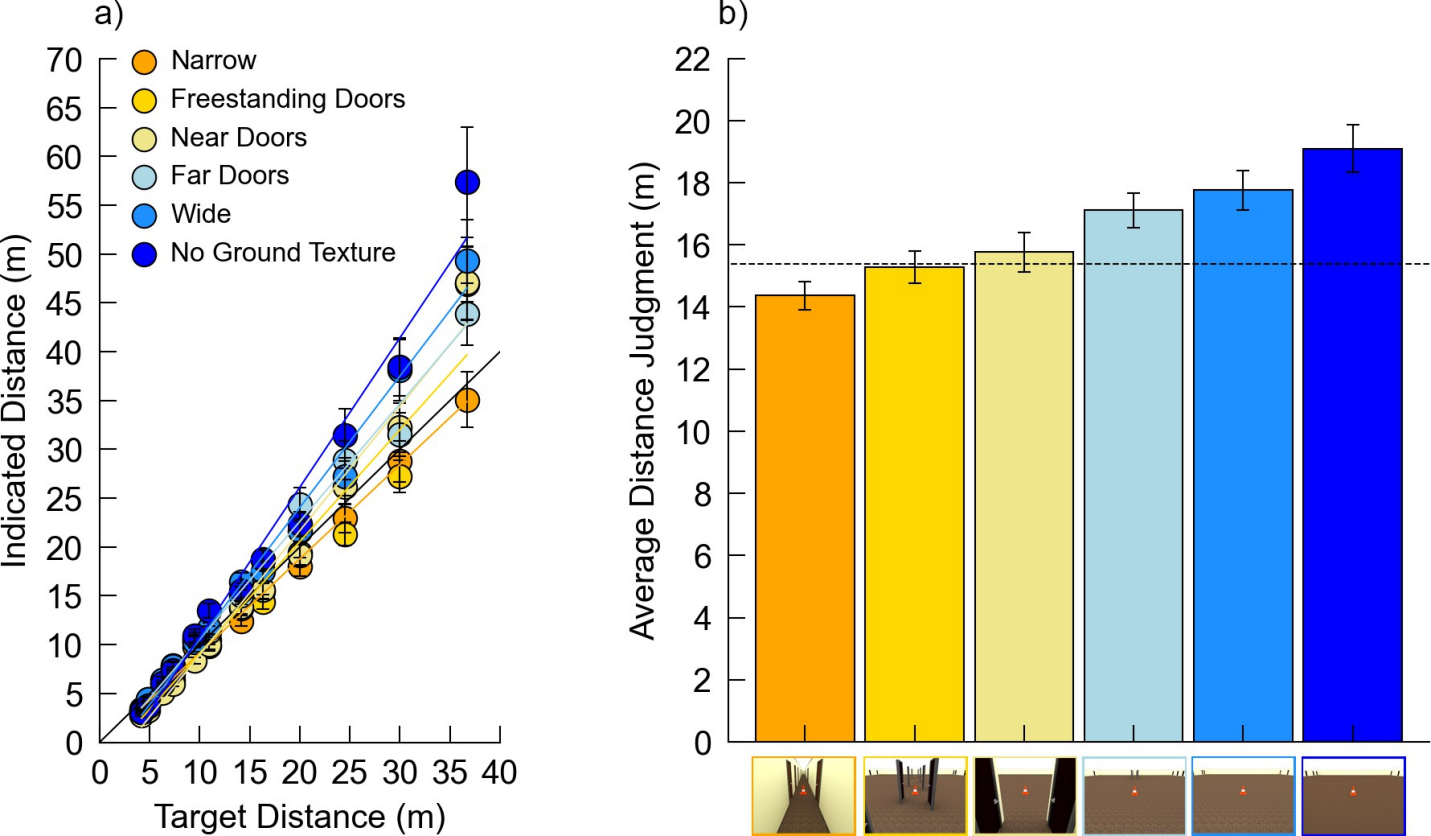
Data from Experiment 4. Error bars denote +/- 1 standard error of the mean. (a) Plot of the indicated distance over the actual distance for all conditions. (b) Average distance judgment for each room condition. The dotted line indicates the actual average target distance. Images on the x-axis depict the room width conditions from Fig 8.

192 trials fell outside 2.5 SD from the mean for each combination of room condition and target and were removed (2.64% of data). No participants were removed for having less than one-third of the trials remaining in any one condition, and all 101 participants were included in the final analysis. Fig 9A plots the indicated distance over the actual distance for each combination of room condition and distance and Fig 9B shows the average distance judgment for each condition (Fig 9). Contrary to the previous studies, the judgments overshot the physical (rendered) distances, but only by 0.27% on average.

As in the previous two experiments, the data were analyzed using linear mixed models via maximum likelihood variance estimation, with Satterthwaite’s method for degrees of freedom correction. *Room* and *Distance* were set as fixed factors and *Subject* as a random factor for a repeated measures design. The interactions between *Room* and *Distance* were also included in the model. Replicating the results of Experiments 2 and 3, the results indicated a main effect of *Room* (*F*[5,6979.0] = 20.51, *p* < 0.001, *η*_*p*_^*2*^ = 0.01), a main effect of *Distance* (*F*[11,6979.3] = 584.10, *p* < 0.001, *η*_*p*_^*2*^ = 0.48), and a significant *Room* x *Distance* interaction (*F*[55,6979.1] = 3.35, *p* < 0.001, *η*_*p*_^*2*^ = 0.03). Pairwise comparisons (at alpha = 0.05 with a Holm correction) are listed in Table 7 (Table 7). The coefficient estimates and standard errors for all conditions are included in S11 File.

**Table 7:** Pairwise comparisons and absolute mean differences for Experiment 4.

	Narrow	Freestanding Doors	Near Doors	Far Doors	Wide	No Ground Texture
**Narrow**		0.91	1.40	2.75	3.40	4.73
**Freestanding Doors**	0.112		0.49	1.84	2.49	3.82
**Near Doors**	0.024	0.428		1.35	2.00	3.33
**Far Doors**	<0.001	0.019	0.108		0.65	1.99
**Wide**	<0.001	<0.001	0.004	0.404		1.33
**No Ground Texture**	<0.001	<0.001	*<*0.001	0.001	0.061	

The pairwise *p*-values and their associated absolute mean differences in meters for Experiment 4. The *p-*values populate the lower left cells, and the absolute mean differences populate the upper right cells. Significance is determined at alpha = 0.05 with a Holm correction.

### 5.3 Discussion

As in the previous three experiments, the main effect of *Room* indicates a difference in response bias across the various room conditions, and the *Room* x *Distance* interaction indicates that *Room*-related response differences are associated with differences in response sensitivity, becoming more pronounced as target distance increases. Replicating the primary room width effect, responses in the *Wide* condition were significantly larger than those in the *Narrow* condition. Three conditions tested the role of doors in various regions within the environment: *Near Doors*, *Far Doors*, and *Freestanding Doors*. Of these conditions, only *Freestanding Doors* yielded statistically similar responses to the *Narrow* condition, suggesting that placing objects (doors) just in the farther or just in the nearer reaches of the scene, with minimal occlusion of the environment, is not sufficient to elicit the shorter distance judgments associated with the *Narrow* rooms.

Our occlusion manipulations in the previous experiments impacted several aspects of the environment: among other possible features, they introduced objects (walls or doors) into the scene, disrupted the ground plane texture, and made more distant regions of the environment less visible. The *Far Doors* condition in Experiment 4 was intended to test the role of introducing objects near the target in a way that involved very little occlusion of the environment. Responses in the *Far Doors* condition did not differ from the *Wide* pattern, suggesting that the presence of objects near the target, per se, is not the driving factor underlying the width effect. The *No Ground Texture* condition, meanwhile, addressed the role of disrupting the ground texture, and interestingly, removing the ground plane texture entirely had no reliable effect relative to the *Wide* response pattern. Thus, altering the fine-grained texture gradient, per se, appears to play little role in this paradigm. This is only one possible way to disrupt the ground plane texture, however—larger-scale texture or color discontinuities on the ground surface (with or without occlusion of more distant parts of the environment) may yet produce some effect. Further research is required to resolve this issue.

## 6. General discussion

Across 4 experiments involving 452 participants judging targets at distances up to 37 m, we have shown that as deep indoor spaces become wider, targets are judged as farther away. Average judgments for the farthest targets in a 40 m wide room were between 16–33% larger than for the same target distances seen in a 1.5 m hallway. The room width effect holds even when cues to the egocentric distance of the target are held constant, highlighting the importance of more peripheral contextual information. Further, obscuring the fine-grained ground plane texture is not primarily responsible for this effect.

At a broad level, the presence of occlusion in the scene seems to be driving the width effect. Occlusion within the nearby environment elicits the *Narrow* pattern, even when the farther reaches of the environment remain visible; likewise, occlusion within the far environment elicits the *Narrow* pattern, even when the nearby regions remain visible. Consistent with this view, the *Freestanding Doors* conditions involved substantial occlusion by the nearby doors, even though portions of the nearby and farther reaches remained visible, and accordingly yielded the *Narrow* response pattern. In sum, we have attempted to separately assess several environmental features associated with occlusion, and to this point, shorter distance judgments seem to be elicited when relatively large regions of the environment are rendered less visible by objects.

Egocentric distance information available from the nearby ground plane can be used as a reference for scaling relative distance cues in real-world environments [17, 40, 49–51]. When viewing pictures, this role is likely diminished, not only because the nearby ground plane is truncated by the border of the picture itself, but also because many of the egocentric distance cues available when viewing a picture provide a signal that the image is flat. Earlier, we suggested that observers might rely on more global scene information to scale relative distance cues, e.g., information that specifies the overall depth of the environment [47]. This predicts that manipulation of features even in the farther regions of space (outside the nearby ground plane) can influence egocentric judgments, and this is exactly what we have found. In particular, our work has shown that the presence of walls in the farther reaches of the environment centered in the image is associated with shorter distance judgments. Importantly, this global image feature mechanism need not be mutually exclusive with the nearby ground plane reference mechanism [50]. Indeed, we assume that when egocentric distance cues are abundant and highly reliable (e. g., in real environments, within 6 m of the observer and with binocular vision), the visual system likely relies heavily on these cues to scale the relative distance cues.

The visual information underlying extraction of mean depth in pictured scenes remains poorly understood, but a variety of global image features no doubt plays a role (e.g., features associated with openness and ruggedness) [52, 53]. Indeed, so many environmental features covary with the size and shape of the setting that Sedgwick and Gillam [54] have suggested that perception of the spatial aspects of pictured scenes may transcend a modular, cue-based understanding (See also Gibson [49]).

Although we did not attempt to measure this directly, it is possible that the perceived slant of the ground plane varied in correlation with the distance judgments, such that when distances were systematically judged to be smaller, the ground plane slant was perceived to be tilted upwards toward the far end of the scene. This could be an alternate explanation of the width effects, if changes in the perceived ground slant were the causative mechanism rather than direct changes in mean depth. It is not obvious how some of our manipulations that influenced distance judgments may have impacted perceived ground slant (e.g., isolated doors in the intermediate-to-far distances). Nevertheless, if the perceived spatial layout were globally rescaled toward smaller distances, resulting in a smaller mean depth, the ground plane should appear tilted, assuming that the perceived eye height and angular directions remained otherwise constant [50]. Thus, mean depth and perceived ground slant are likely to be linked, but future research is required to determine if there is a direction of causality in this linkage.

Distance judgments made in real-world indoor settings are sometimes *larger* than those of the same physical distances in outdoor settings [18, 19, 55, 56]. Although it is plausible that the constellation of egocentric and exocentric distance cues available in indoor environments is generally enriched relative to outdoor settings, the cue bases of this result are not known, so it is not clear whether environment size or shape play a role. Our results with pictured scenes indicate that narrower settings are associated with *smaller* distance judgments than wider environments. There are many differences between viewing pictures of scenes and directly viewing the scenes while being immersed in them, as authors have frequently noted [2, 4, 11, 28, 57–60]. More research is required to determine the extent to which the current results involving pictured stimuli might generalize to real-world contexts. Manipulating the width of large, real environments presents considerable logistical challenges. Virtual environments would provide a convenient means of exploring these issues, as virtual environments afford fine-grained control over the size, shape and contents of the setting and allow the observer to be more fully immersed in the scene. Nevertheless, our results indicate a robust effect of environment width on egocentric distance judgments in pictured settings. Given the wide variety of electronic devices available and the prevalence of pictured scenes in electronic content, this result has broad applicability for understanding how humans process spatial relationships in pictures.

## Supporting information

S1 File Raw data for Experiment 1. (CSV)

S2 File Raw data for Experiment 2. (CSV)

S3 File Raw data for Experiment 3. (CSV)

S4 File Raw data for Experiment 4. (CSV)

S5 File Coefficient estimates for the 6m depth condition in Experiment 1. (CSV)

S6 File Coefficient estimates for the 10m depth condition in Experiment 1. (CSV)

S7 File Coefficient estimates for the 40m depth condition in Experiment 1. (CSV)

S8 File Coefficient estimates for the depth comparison analysis in Experiment 1. (CSV)

S9 File Coefficient estimates for Experiment 2. (CSV)

S10 File Coefficient estimates for Experiment 3. (CSV)

S11 File Coefficient estimates for Experiment 4. (CSV)
